# A Study Comparing the Effects of Targeted Intra-Arterial and Systemic Chemotherapy in an Orthotopic Mouse Model of Pancreatic Cancer

**DOI:** 10.1038/s41598-019-52490-1

**Published:** 2019-11-04

**Authors:** Melika Rezaee, Jing Wang, Mehdi Razavi, Gang Ren, Fengyan Zheng, Ahmed Hussein, Mujib Ullah, Avnesh S. Thakor

**Affiliations:** 1 Interventional Regenerative Medicine and Imaging Laboratory, Stanford University School of Medicine, Department of Radiology, Palo Alto, California, 94304 USA; 2 Chicago Medical School, Rosalind Franklin University, North Chicago, Illinois 60064 USA

**Keywords:** Cancer models, Targeted therapies

## Abstract

Systemic chemotherapy is the first line treatment for patients with unresectable pancreatic cancer, however, insufficient drug delivery to the pancreas is a major problem resulting in poor outcomes. We evaluated the therapeutic effects of targeted intra-arterial (IA) delivery of gemcitabine directly into the pancreas in an orthotopic mouse model of pancreatic cancer. Nude mice with orthotopic pancreatic tumors were randomly assigned into 3 groups receiving gemcitabine: systemic intravenous (IV) injection (low: 0.3 mg/kg and high: 100 mg/kg) and direct IA injection (0.3 mg/kg). Treatments were administered weekly for 2 weeks. IA treatment resulted in a significantly greater reduction in tumor growth compared to low IV treatment. To achieve a comparable reduction in tumor growth as seen with IA treatment, gemcitabine had to be given IV at over 300x the dose (high IV treatment) which was associated with some toxicity. After 2 weeks, tumor samples from animals treated with IA gemcitabine had significantly lower residual cancer cells, higher cellular necrosis and evidence of increased apoptosis when compared to animals treated with low IV gemcitabine. Our study shows targeted IA injection of gemcitabine directly into the pancreas, via its arterial blood supply, has a superior therapeutic effect in reducing tumor growth compared to the same concentration administered by conventional systemic injection.

## Introduction

In the United States, approximately 55,440 patients are diagnosed with pancreatic cancer each year^1^. Although pancreatic cancer is currently the fourth leading cause of cancer related death^2^, it is expected to rise and become the second leading cause of cancer related death in the United States by 2020^3^. The majority of pancreatic cancer tumors (85%) are adenocarcinomas arising from the ductal epithelium (pancreatic ductal adenocarcinoma; PDAC)^1^. As most patients are diagnosed late and often have advanced disease, the overall 5-year survival rate for this disease is only 8%^1^.

The development of effective therapies for pancreatic cancer has been slow with only modest improvements over the past few years. The main treatment approaches for pancreatic cancer include systemic chemotherapy and/or surgery^4^. Although surgical resection of the primary tumor offers the possibility for a cure when the disease is localized, which occurs in only 15–20% of patients, the remaining 80–85% of patients present late with advanced or even metastatic disease^5–8^. Systemic chemotherapy plays a central role in the treatment of pancreatic cancer diagnosed at all stages^9^, and is the first line of treatment for patients with unresectable disease^10^. One of the most widely used chemotherapeutic drugs to treat pancreatic cancer is gemcitabine - a pyrimidine antagonist which blocks the creation of new DNA that can be used as either a single agent or in combination therapy^11^. However, studies have shown that pancreatic cancer is relatively resistant to systemic chemotherapy administration. In part, this is attributed to the tumor’s hypo-vascularity relative to the surrounding parenchyma which results in it having a lower perfusion. In turn, this will limit the ability of any systemically administered drug to penetrate into the tumor^12^. In addition, pancreatic tumors also have a dense stroma surrounding the tumor cells^13^. This stroma, which is composed of pancreatic stellate cells (PSCs), inflammatory and immune cells, endothelial cells and the extracellular matrix (ECM)^14^, not only forms a physical barrier to chemotherapy penetration into the tumor but also interacts with, and promotes the progression of, tumor cells.

Initially it was thought that increasing the dose of chemotherapeutics, like gemcitabine, would overcome these obstacles and enable the effective treatment of pancreatic cancer; especially since the therapeutic effects of such drugs have been shown to be dose-dependent in cell culture studies^15^. However, clinical studies in general have shown that increasing the systemic dose of chemotherapeutics results in a higher incidence of systemic toxicity with often only a marginal increase in therapeutic benefit^15–17^. Hence, to effectively treat tumors like pancreatic cancer, more targeted approaches are needed to ensure that drugs actually reach and penetrate into the tumor. One way to address this issue is to deliver drugs directly into the tumor thereby ensuring that they reach tumor cells in the highest possible concentration. Although percutaneous access directly into the tumor is possible, this will not uniformly distribute a drug throughout the tumor parenchyma. Furthermore, in the case of pancreatic cancer, the tumors are very hard to target given their deep intra-abdominal location and the surrounding vital structures. Hence, an alternative approach is to directly deliver drugs to pancreatic tumors via their arterial blood supply.

Over the past few decades, the ability to intra-arterially (IA) deliver chemotherapeutics directly to tumors using minimally-invasive image guided endovascular approaches has transformed the care of cancer patients. For instance, in patients with liver cancer, IA administration of chemotherapy directly into the tumor via the hepatic arterial blood supply, has shown to significantly improve the 2-year survival of patients from 27% to 63%^18–20^. This approach overcomes the shortcomings of conventional intravenous (IV) administration which includes systemic toxicity, first pass metabolism and non-target delivery. Until recently, IA chemotherapy administration to pancreatic tumors has not been possible, mainly due to the complicated arterial blood supply of the pancreas with its numerous anastomoses^21^. However, advances in both microcatheter technology and imaging equipment have now opened the possibility of efficiently accessing the arterial blood supply of the human pancreas. Hence, there is now an urgent need to develop techniques to deliver therapeutics directly to the pancreas, via its arterial blood supply, in small animal orthotopic models of pancreatic cancer to enable the optimization and testing of new therapies. Orthotopic models of tumors are commonly used in translational research to evaluate the antitumor efficacy of chemotherapeutics^22^. Advantages of orthotopic models include the use of real human tissue which can be then implanted at appropriate anatomical sites^23^. As such, these tumor cells will be exposed to the correct microenvironment where they will then grow and metastasize thereby replicating as close as possible what would occur in human patients^24^.

Hence, in the present study, we will use an orthotopic pancreatic tumor mouse model which we will create using immunocompromised mice and AsPC1 cells (i.e. a well-established human pancreatic cancer cell line). Recently, we developed a novel and reproducible microsurgical technique to deliver therapeutics directly into the pancreas of a rat, via its arterial blood supply, without inducing physical (i.e. bleeding) or biochemical (i.e. pancreatitis) damage to the gland^25^. Using a modified version of this technique, we will now evaluate the therapeutic benefits and toxic side effects of gemcitabine when it is administered in mice with orthotopic pancreatic tumors, either by direct IA or systemic IV injections.

## Results

### Validation of our IA technique for delivery for therapeutic delivery directly into the pancreas

**Figure 1:**
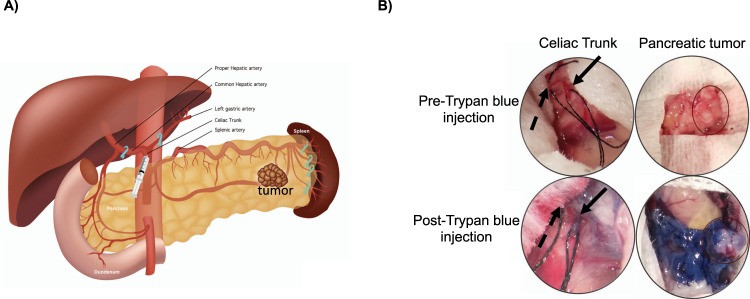
Schematic representation of intra-arterial delivery to the pancreas. (**A**) Schematic representation of the arterial blood supply to the pancreas and the ligation sites used to isolate the tumor in the tail of pancreas (image by Amy Thomas, web and graphic designer at Stanford University). (**B**) Pre- and post -trypan blue injection into the celiac trunk demonstrating selective delivery into the tumor implanted within the pancreas. The black arrow shows the celiac trunk and the dashed arrow shows a ligature around the hepatic artery.

Following IA injection of trypan blue dye directly into the celiac trunk (Fig. 1A), we demonstrated that the reversible ligation of the hepatic, splenic and gastric arteries resulted in redistribution of blood flow, and hence trypan blue dye, into the pancreas. Indeed, we noted that orthotopic pancreatic tumors were stained blue with no evidence of any staining in extra-pancreatic organs (n = 4) (Fig. 1B). Furthermore, there was no biochemical evidence of pancreatic injury or histological evidence of pancreatic inflammation in animals following IA injection of gemcitabine. The average amylase and lipase levels at baseline measured: 1220 ± 273 U/L and 236 ± 33 U/L and two days post-treatment: 1330 ± 211 U/L and 269 ± 62 U/L, respectively (P > 0.05). The reference range for normal amylase and lipase levels in rodents are < 1400 and 176–322 U/L, respectively.

### Determination of the optimal dose of gemcitabine for IA administration

**Figure 2:**
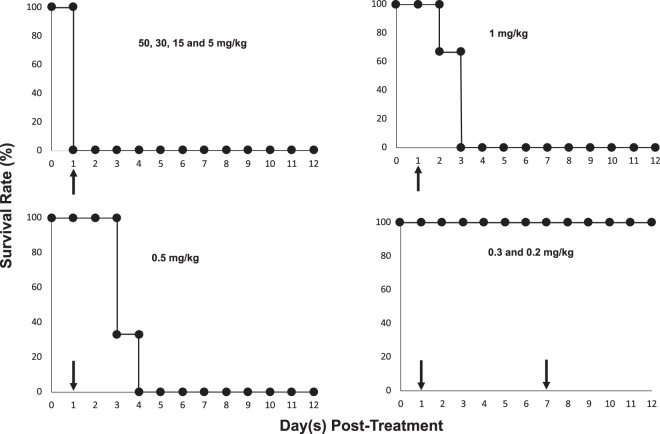
Determination of intra-arterial chemotherapy concentration Different concentrations of gemcitabine (0.2–50 mg/kg) were injected intra-arterially (n = 3 for each dose) with each animal survival monitored for two weeks. Black arrows represent treatment days.

To determine the optimal concentration of gemcitabine which can be given IA to mice, we administered gemcitabine at concentrations from 0.2 to 50 mg/kg once a week for two consecutive weeks and monitored the morbidity and mortality of animals. Our data shows that the mortality rate of animals treated with gemcitabine at concentrations greater than 0.5 mg/kg was 100% after the drug was given IA directly into the pancreas. Indeed, all animals treated IA with 50, 30, 15 and 5 mg/kg gemcitabine died within 24 hours. Of the animals treated IA with 1 mg/kg gemcitabine, all animals died within 72 hours and of those treated IA with 0.5 mg/kg gemcitabine, all animals died within the first week after treatment. However, all animals treated IA with gemcitabine at 0.3 and 0.2 mg/kg survived two doses given consecutively each week (Fig. 2).

### The effect of IA vs IV administration of gemcitabine on tumor growth

**Figure 3:**
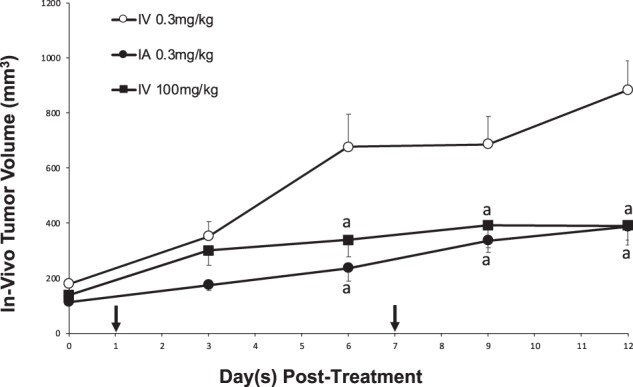
*In-vivo* tumor volume Tumor size was monitored every 3 days using ultrasound in groups treated with IV 0.3 mg/kg, IV 100 mg/kg and IA 0.3 mg/kg. P < 0.05 a: vs IV 0.3 mg/kg; b: vs IV 100 mg/kg. Black arrows represent treatment days.

**Figure 4:**
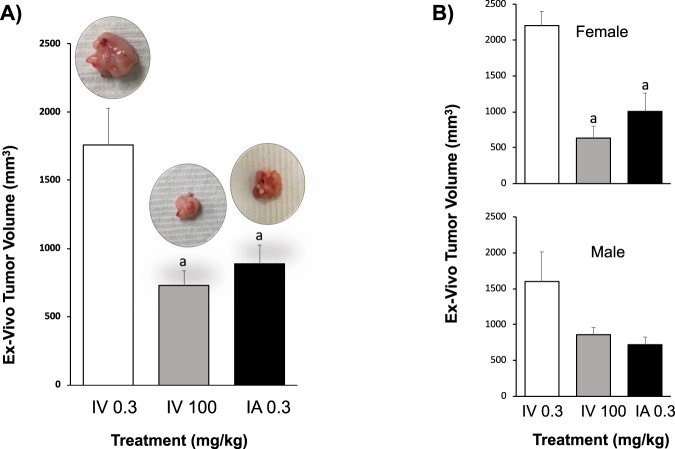
*Ex-vivo* tumor volume. (**A**) *Ex-vivo* tumor volume measurements in groups treated with IV 0.3 mg/kg, IV 100 mg/kg and IA 0.3 mg/kg. (**B**) *Ex-vivo* tumor volume measurements in female and male groups separately. P < 0.05 a: vs IV 0.3 mg/kg; b: vs IV 100 mg/kg.

To determine the effect of chemotherapy administration on pancreatic tumor growth, the *in vivo* tumor volume was calculated every 3 days using ultrasound. In animals treated with low IV gemcitabine, there was a steady increase in tumor volume over two weeks (Baseline: 171 ± 17 mm^3^, Week 1: 621 ± 116 mm^3^, Week 2: 829 ± 105 mm^3^). In contrast, animals treated with IA gemcitabine at the same concentration resulted in a significantly attenuated increase in tumor volume over two weeks (Baseline: 114 ± 11 mm^3^, Week 1: 236 ± 48 mm^3^, Week 2: 388 ± 66 mm^3^) when compared to low IV gemcitabine (P < 0.05). Indeed, the beneficial effect of IA gemcitabine was similar to that attained when gemcitabine was given IV (high) at over 300x the dose (Baseline: 143 ± 15 mm^3^, Week 1: 402 ± 73 mm^3^, Week 2: 392 ± 44 mm^3^; P > 0.05) (Fig. 3). At the end of two weeks of treatment, all tumors were harvested and measured *ex vivo*. Again, similar results were obtained with larger tumors in the low IV gemcitabine group (1,759 ± 268 mm^3^) and significantly smaller tumors noted in both the IA and high IV gemcitabine groups (889±137 mm^3^ and 731 ± 106 mm^3^ respectively; P < 0.05) (Fig. 4A). Two animals treated with high IV gemcitabine developed signs of systemic toxicity as demonstrated by jaundice (n = 1) and ascites (n = 2) at the study end point. There was no significant difference in the average weight change between all experimental groups over the duration of the experimental protocol: low IV gemcitabine treatment: −0.63 ± 0.46 g, high IV gemcitabine treatment: −0.12% ± 0.34 g and IA gemcitabine: −1.28 ± 0.45 g (P > 0.05).

Given that each experimental group contained 3 male and 3 female animals, we also performed a subset analysis examining if there was any difference in the responses based on sex. Our results demonstrated that while both male and female animals demonstrated a decreased tumor growth when treated with IA and high IV gemcitabine, compared to low IV gemcitabine, the difference was only statistically significant in females; however, this analysis is limited in its power given that there were only 3 animals per group (Fig. 4B).

### Histological and Immunohistochemical analysis of pancreatic tumor tissue

**Figure 5:**
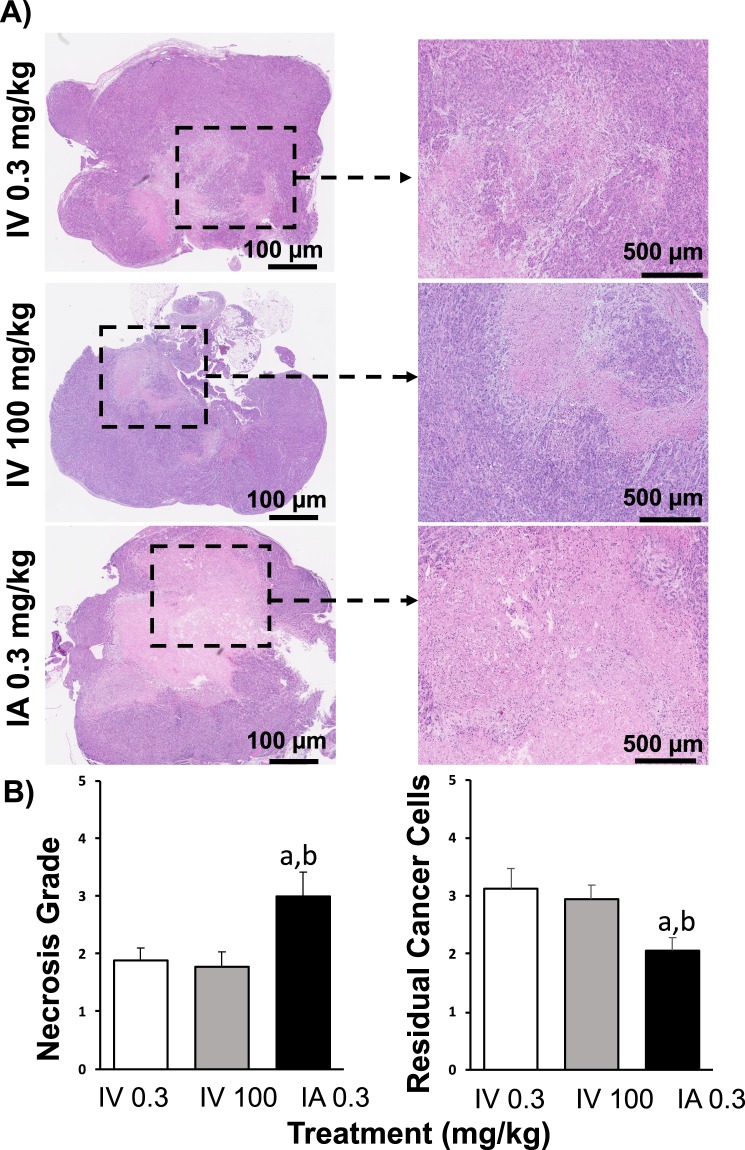
H&E staining of pancreatic cancer. (**A**) Representative micrographs of H&E stained histological sections of orthotopic pancreatic tumors treated with IV 0.3 mg/kg, IV 100 mg/kg and IA 0.3 mg/kg. (**B**) Graphs represented the necrosis grade and residual cancer cells in groups treated with IV 0.3 mg/kg, IV 100 mg/kg and IA 0.3 mg/kg. P < 0.05 a: vs IV 0.3 mg/kg; b: vs IV 100 mg/kg.

**Figure 6:**
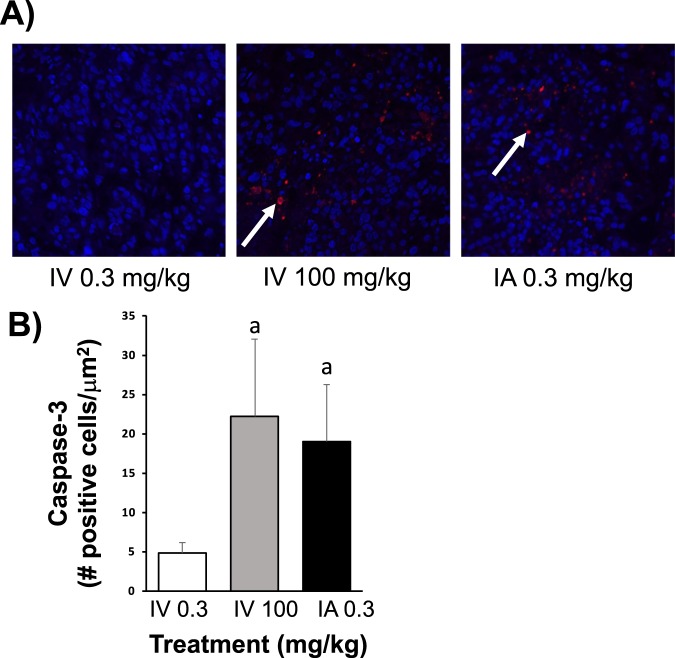
Immunohistochemistry staining of pancreatic cancer. (**A**) Representative micrographs of cleaved caspase-3 (apoptosis biomarker) immunohistochemistry staining sections of orthotopic pancreatic tumors treated with IV 0.3 mg/kg, IV 100 mg/kg and IA 0.3 mg/kg. (**B**) Cleaved caspase-3 staining quantification of tumor tissues treated with IV 0.3, IV 100 or IA 0.3 mg/kg gemcitabine. P < 0.05 a: vs IV 0.3 mg/kg; b: vs IV 100 mg/kg.

Animals treated with IA gemcitabine showed significantly larger regions of necrosis within tumors (grade: 3.0 ± 0.4) when compared to tumors treated with low (grade: 1.8 ± 0.2) and high (grade: 1.8 ± 0.3) IV gemcitabine (P < 0.05; Fig. 5). A similar pattern was seen with the residual number of cancer cells, with the IA gemcitabine group having significantly less cancer cells (grade: 2.1 ± 0.2) compared to tumors treated with low (grade: 3.1 ± 0.4) and high (grade: 3.0 ± 0.2) IV gemcitabine (P < 0.05; Fig. 5). In addition, there was a significantly higher expression of cleaved caspase-3 in tumors treated with IA gemcitabine (19.0 ± 7.2 positive cells/μm^2^) and high IV gemcitabine (22.2 ± 9.8 positive cells/μm^2^) when compared to tumor samples from animals treated with low IV gemcitabine (4.8 ± 1.3 positive cells/μm^2^; P < 0.05; Fig. 6).

## Discussion

In the present study, we demonstrated that IA administration of gemcitabine directly into the pancreas decreased the growth of pancreatic tumors relative to conventional systemic IV administration at the same dose. In order for IV administration to achieve the same effect as IA administration, gemcitabine needed to be administered intravenously at over 300 times the concentration that was given IA to animals; furthermore, at these levels, this was associated with some animals developing evidence of systemic toxicity as demonstrated by jaundice and ascites. Our data also shows that our novel technique for IA administration of therapeutics directly into the pancreas in mice is feasible with no evidence of pancreatic trauma or injury, as evidenced by no increase in amylase or lipase following surgical intervention and no adverse symptoms (i.e. changes in behavior, feeding patterns, posture and activity level). Furthermore, IA administration of gemcitabine resulted in increased evidence of both apoptosis (i.e. an increased expression of cleaved caspase 3) and necrosis in pancreatic tumor samples compared to animals which received same dose IV, thereby demonstrating that the effects of gemcitabine on the tumor are enhanced following targeted delivery.

Patients with early stage pancreatic cancer is usually asymptomatic, and the majority of patients become symptomatic when the tumor becomes locally invasive or metastasizes to other organs^6–8^. Unfortunately, treatment options are limited in the advanced stages of pancreatic cancer and surgical resection is not a feasible option. In patients with unresectable pancreatic cancer, chemotherapy is the primary treatment^10^, however, it only extends life for a few months and is associated with significant systemic toxicity^9^. Gemcitabine is one of the common chemotherapeutic agents used in the treatment of pancreatic cancer^11^. Gemcitabine is a fluorinated cytosine analog prodrug that is transported into cells via a nucleoside transporter; and then it is either phosphorylated leading to its activation or deaminated leading to its elimination^11^. There is a difference between the efficacy of gemcitabine *in vitro* vs *in vivo*, with studies showing more potent tumor cell line response to gemcitabine compared to *in vivo* pancreatic adenocarcinoma models^26–30^, presumably due to cell culture studies failing to inadequately represent the complex *in vivo* microenvironment and phenotypic characteristics of pancreatic cancer. These results are further confirmed in clinical studies which have also demonstrated that pancreatic tumors have significant chemoresistance to gemcitabine mainly due to (i) the presence of very dense and poorly vascularized fibrotic envelopes that surround the tumors making them almost impenetrable by drugs^31^ and (ii) robust multidrug resistance mechanisms, such as the toxin efflux enzyme system glycoprotein (P-170), which are upregulated in tumor cells and result in the rapid clearance and thus elimination of drugs^32^. However, one way to facilitate the penetration of drugs like gemcitabine into tumors, as well as reduce their loss through efflux systems, is to ensure that they are delivered to tumors in high concentrations^33^. Unfortunately, this cannot be achieved using conventional IV administration as the amount of drug which would need to be injected would be associated with profound systemic/toxic side effects, which in the case of gemcitabine includes cytopenia, nausea and bone marrow suppression^34^. However, by delivering drugs directly to tumors, via their arterial blood supply, we can overcome this problem and ensure that the tumor will receive the drug at high enough concentrations for it to be effective, with minimal systemic exposure^15^; together this will facilitate patient recovery, reduce hospital stay and improve patient outcome^18^.

The concept of administering chemotherapy directly to tumors via an IA injection, using minimally invasive approaches, has been used with great success for the treatment of primary and secondary hepatic malignancies. Here, multiple studies have shown that using IA chemotherapy there is improved patient outcome while concurrently minimizing damage to the healthy liver parenchyma and surrounding organs^35^. In addition, direct IA administration of chemotherapy has been used to downstage advanced disease such that patients can then receive a curative surgical resection which would have not been possible at their initial presentation^36,37^. Based on similar indications, IA chemotherapy administration directly to pancreatic tumors could therefore potentially be used to either treat tumors or downstage tumors such that non-operative patients could become eligible for surgical resection. Recently, we developed a novel technique to deliver therapeutics to different regions of the rat pancreas, via its arterial blood supply^25^. In the present study, we adapted this microsurgical technique and were able to optimize it for targeted delivery to the pancreas in mouse. Indeed, our results showed that this technique is feasible, reproducible and did not adversely affect the pancreas or surrounding organs; this was supported by our data which showed that following IA Trypan blue injection, we were able to consistently stain the pancreas (and orthotopic pancreatic tumors) with no hematological evidence of pancreatitis. In addition, we were able to perform this technique up to 2 times in the same animal; additional interventions could not be performed without significant mortality given the amount of microdissection that needed to be performed to re-access the celiac artery which was noted to have considerable scar tissue and adhesions surrounding its origin after the second surgical procedure. However, this would not be the case when translated to humans given that the arterial supply to the pancreas can be accessed several times in the same patient using endovascular approaches. In summary, this technique will now lay the foundation for researchers to explore the effects of different therapeutics (both chemical and cellular therapies) directly on the pancreas in mice, while avoiding issues related to non-targeted delivery.

In our *in vivo* studies, we found that 0.3 mg/kg of gemcitabine by IA administration was the highest chemotherapy dose that was tolerated in mice. At higher doses, all animals died as a result of significant pancreatic inflammation as noted on post-mortem examination. Hence, we used 0.3 mg/kg of gemcitabine as our treatment dose for our experimental groups. Although this dose was significantly lower than what has been used in previous small animal studies for IV administration, which range from 2 to 240 mg/kg^38,39^, we found that when this dose was given IA to mice with orthotopic pancreatic tumors there was a reduction in tumor growth when compared to the same dose administered IV. Indeed, in order to achieve the same therapeutic effect as IA administration, gemcitabine needed to be administered systemically by IV injection at over 300 times the dose thereby demonstrating the efficacy of this approach for the treatment of pancreatic cancer. While there was no significant difference in the tumor volumes in the IA 0.3 mg/kg and IV 100 mg/kg groups, the IV 0.3 mg/kg group did have a slightly higher starting tumor volume despite all animals being blindly randomized to each experimental group at Day 0. Nevertheless, the overall trend of the growth of tumors treated with IA 0.3 mg/kg and IV 100 mg/kg was markedly reduced compared to animals treated with IV 0.3 mg/kg. This marked reduction in tumor proliferation was evident over the experimental protocol, especially at the later time points. Tumor responses to chemotherapy were also assessed histologically, looking specifically at apoptosis, necrosis and residual pancreatic cancer cells in tumors treated with gemcitabine. Tumors treated with IA gemcitabine demonstrated a higher grade apoptosis (i.e. increased expression of cleaved caspase-3 staining) compared to tumors treated with the same dose of IV gemcitabine. These results are supported by the assumption that a greater amount of gemcitabine is delivered to tumors following IA injection and evidence that gemcitabine is able to induce apoptosis in tumors^40–42^. Correspondingly, there was a lower percentage of residual cancer cells in these tumors compared to those treated with IV gemcitabine (at both and high and low doses). Interestingly, tumors treated with IA gemcitabine showed an increase in necrosis relative to tumors treated with IV gemcitabine, despite their smaller size. This is in contrast to several studies which suggest that necrosis is seen predominantly in larger tumors, given the greater amount of intra-tumoral hypoxia present, which, in turn results in tissue necrosis^43^. One explanation for this could be that gemcitabine has been shown in some studies to inhibit angiogenesis, which in turn could then lead increased tumor hypoxia and hence necrosis^44^.

Given the increased awareness of differences in sex in guiding translational research^45,46^, we performed a subset analysis on our results given that each of our experimental groups contained male (n = 3) and female (n = 3) animals. We found that while the same trend was seen, with IA being more efficacious than IV administration, this effect reached statistical significance only in the female group. The difference in drug response in males vs females could be linked to sex-related difference at the genetic levels^47^. For example, in one study Yamamoto H *et al*. showed that combination therapy of paclitaxel and carboplatin improved progression-free survival rate in female patients more than in male patients with non-small cell lung cancer^48^. In another study, Wei Q *et al*. explained the sex-related response to drug therapy might be caused by the lower level of DNA repair in females^49^. and hence might affect the ability of tumor cells in females to survive following administration of cytotoxic anticancer drugs. Although our result should be interpreted with caution given the small number of animals in each group, future studies will aim to increase the power of this study using groups of different sexes with a larger sample size.

In conclusion, this study has shown that targeted IA delivery of gemcitabine directly to the pancreas decreases tumor growth significantly compared to the same dose of drug given systemically. To achieve the same effect, the drug would need to be given at 300 times the dose systemically which was associated with significant side effects in several animals. In addition, this novel technique to deliver chemotherapy to the pancreas in mice, via its arterial blood supply, is safe and feasible with no evidence of physical or biochemical damage. Using this platform, combination chemotherapies (i.e. 5-FU, cisplatin and paclitaxel) as well as with other therapeutic approaches (i.e. cellular therapies, nanoparticle and microbubble based platforms) can now be explored.

## Methods

### Animals

Male and female immunocompromised athymic nude (nu/nu) mice at 6 weeks of age were purchased from Jackson Laboratory and housed at Stanford’s Canary Center Animal Care Services. All animals were housed under standard conditions (12 hr:12 hr light:dark cycles temperature at 21.8 °C and humidity at 50%) and had free access to food and water. All studies were approved following review by Stanford’s Administrative Panel on Laboratory Animal Care, and all methods detailed in this manuscript were performed in accordance with these guidelines and regulations.

### Development of mice models of orthotopic pancreatic cancer

AsPC1 cells (ATCC, Manassas, VA, USA; a 62-year-old Caucasian female with pancreatic adenocarcinoma) were grown in RPMI 1640 medium supplemented with 10% Fetal Bovine Serum and 1% penicillin and streptomycin (Life Technologies, Grand Island, NY, US). Cells were incubated in humidified atmosphere at 37 °C and 5% CO_2_, with the culture media changed every 3 days. Initially, pancreatic cancer xenograft models were created using 1.5 M AsPC1 cells which were placed in 100 µl of matrigel and then subcutaneously injected into the flank of 6-week-old nude mice^50^. After three weeks, xenograft tumors reached 10 mm in size at which point they were removed from each animal and cut into small blocks (2 × 2 × 2 mm^3^). Each tumor block was then implanted into the pancreas of a new group of nude mice, as previously described^50^. In brief, a 10 mm vertical skin incision was made on the left side of the upper abdomen of a recipient nude mouse. The peritoneum was then carefully opened, and the pancreas exposed. A single block of tumor was then carefully implanted into the body of the pancreas. The pancreas was then placed back into the abdominal cavity and abdominal wall closed using a 5–0 absorbable suture and surgical staples^51^. Following the surgery, animals (n = 6 for each treatment group) were observed on heat pad for 30 minutes before returning to their cage. The surgical wound was monitored daily for any signs of wound infection, bleeding or wound dehiscence or other post-procedure complications (i.e. changes in their weight, behavior, feeding patterns, posture and activity level). Staples were removed on postoperative day 7–10 with sterile surgical staple removal.

### Study design

After orthotropic tumor implantation, tumor growth was monitored using ultrasound scanner (Siemens Healthcare, Issaquah, WA) every 3 days. Once orthotopic tumors reached 6 mm, which usually was 2–3 weeks after tumor implantation, animals were randomly allocated into 3 groups (n = 6 per group; n = 3 males and n = 3 females): Group 1 = systemic intravenous (IV) injection of gemcitabine at 0.3 mg/kg (low); Group 2 = systemic IV injection of gemcitabine at 100 mg/kg (high); and Group 3 = direct intra-arterial (IA) injection of gemcitabine at 0.3 mg/kg. Each animal received treatments at the start of week 1 and 2. Tumor volumes were calculated based on their height, width and length measurements which were taken every 3 days using ultrasound. Animals were humanely euthanized at the end of week 2, at which point the tumor dissected free from the pancreas and measured using a calibrated caliper (CRK Precision). In addition, the liver, spleen and peritoneum were fully examined for any signs of metastasis. Throughout the experimental protocol, all animals were monitored for signs (i.e. ascites and jaundice) of disease progression.

### Gemcitabine concentration determination

Previous studies have shown that the therapeutic concentration of gemcitabine when given systemically to small animal models is in the range of 2–240 mg/kg^38,39^. To determine a concentration of gemcitabine which would be safe to give IA directly into the pancreas, in nude mice, a dose study was performed in which 24 nude mice (n = 3 per group) were given different gemcitabine concentrations (50, 30, 15, 5, 1, 0.5, 0.3 and 0.2 mg/kg) once a week for two weeks. The morbidity and mortality of all animals were then evaluated.

### Routes of chemotherapy administration

For IV administration, animals were anesthetized using isoflurane anesthesia (2%) and gemcitabine (0.3 or 100 mg/kg) dissolved in 100μl of heparinized saline was injected into the tail vein using a 28-gauge needle (n = 6 for each group). For IA administration, we modified a technique previously developed in our lab for the delivery of therapeutic agents directly into the rat pancreas via its arterial blood supply^25^. In brief, animals were anesthetized using isoflurane anesthesia (2%), and the abdomen then prepped and draped in the usual sterile fashion. All surgical procedures were performed using sterile technique. The abdominal wall was then opened with a longitudinal 3 cm midline incision to expose the medial border of the liver, stomach, pancreas and duodenum. Any intra-abdominal organs (i.e. loops of bowel, stomach, liver and spleen) which were mobilized outside of the abdomen, or exposed during surgery, were covered with a moist gauze. Using careful microdissection, the abdominal aorta, celiac artery, common hepatic artery and splenic artery were then exposed. Next, 7–0 nylon sutures were looped (but not ligated) around the common hepatic, splenic and gastric arteries. The celiac artery was then cannulated with a 36-guage beveled nanofil needle (World Precision Instrument, FL, USA) with care taken not to traverse the vessel. Prior to the start of injection, the sutures were closed around the common hepatic and splenic arteries (when visible the gastric artery was also ligated); these sutures were then immediately opened following injection. The maximum volume injected into each animal was 100 µl over 30 sec. At the end of the injection, the needle was removed from the celiac artery and light pressure applied to the arteriotomy site using a cotton Q-tip for at least 3 min to achieve hemostasis. The intra-abdominal organs were then returned back to the abdomen and the abdominal wall closed using a 5–0 suture and surgical staples. Following the surgery, animals were observed on heat pad for 30 minutes before returning to their cage. Animals were monitored daily following surgical intervention for any signs and symptoms of post-procedure complications (i.e. changes in their weight, behavior, feeding patterns, posture and activity level).

### Validation of our intra-arterial microsurgical technique

Initial validation of our modified microsurgical technique was undertaken in nude mice with orthotopic pancreatic tumors (n = 4). In these experiments Trypan blue was injected as a surrogate marker of any chemotherapeutic agent directly into the celiac trunk following reversible ligation of the common hepatic and splenic arteries (Fig. 2A,B). At the end of the injection, the biodistribution of the dye was determined by examining the intra-abdominal organs (pancreas, stomach, bowel, liver and spleen) for any staining. In addition, 100 µl of blood was collected from all animals, via their submandibular artery, to measure serum markers of pancreatitis (i.e. amylase and lipase) on post-procedure day 2.

### Histology and immunohistochemistry

All experimental animals (n = 6 per each treatment group) were euthanized at the end of the study at which time tumors were harvested and fixed with 10% buffered formalin, embedded in paraffin and sectioned at three different levels: superficial, midway and central. Sections were cut at 5 µm thickness using a HM 355 S automatic microtome (ThermoFisher Scientific). All samples were stained with hematoxylin and eosin (H&E) at Histo-Tech Laboratory (Hayward, CA) and sections were evaluated for necrosis using a light microscope^52^. The tumor response was assessed into five grades based on the percentage of residual cancer cells and necrosis within samples: grade 1; less than 20%, grade 2; between 20–40%, grade 3; between 40–60%, grade 4; between 60–80% and grade 5; greater than 80%, respectively^53^. Each section was evaluated by two examiners who were blinded to the study groups. For immunohistochemistry staining, sections were dewaxed in Clearify^TM^ (American Mastertech), and then rehydrated in alcohol series. Samples were then immersed in 3% hydrogen peroxide for 10 min to suppress endogenous peroxidase activity, followed by heating at 100 °C for 20 minutes in 0.01 M sodium citrate buffer (pH 6.0) to retrieve antigens. Samples were then rinsed in PBS for 5 minutes before being incubated at 4 °C overnight with rabbit polyclonal antibodies to cleaved caspase 3 (Abcam, 1:100). The following day, samples were washed 3 times in PBS with 0.1% triton X-100 (ThermoFisher Scientific) and then incubated with a fluorescent labelled conjugated secondary antibody at room temperature for 1 hour. All the slides were examined under a confocal microscope (Ziess LSM710) at 40x magnification. All 6 tumors from each treatment group were evaluated for histology and immunohistochemistry analysis. For each animal, 3 histological slide sections (n = 3) from different areas of the tumor were evaluated randomly (each tumor slide section were at least 30 µm or more from each other) and final grade for each treatment group was obtained by averaging all the slide sections in each group (n = 18 slides per each group). Using FIJI Image J software, the positive staining within islets was then determined.

### Statistical analysis

All data are presented as the mean ± SEM. Kaplan-Meier survival curves were plotted to determine the optimal gemcitabine concentration for IA injection. Comparison of variables between groups was done with a Two-way ANOVA with repeated measures comparing the effect of time and treatment. Histological results were analyzed using one-way ANOVA. Statistical analysis was performed using Prism Version 7.0d and a significance level of P < 0.05 was used.

## Data Availability

All data supporting findings of this study are available within the article or from the corresponding author upon request.
